# Epilepsy Secondary to a Giant AVM: A Case Report

**DOI:** 10.1155/crvm/5668999

**Published:** 2025-07-23

**Authors:** Emilio García Gómez, Daniela Carolina Pimentel Saona, Juan Romero Valencia, Lenin Sandoval Luna, Cristobal Jeronimo Ortega Arenas, Daniel San-Juan

**Affiliations:** ^1^Epilepsy Clinic, National Institute of Neurology and Neurosurgery, Mexico City, Mexico; ^2^Faculty of Medicine, Benemerita Autonomous University of Puebla, Puebla, Mexico; ^3^Neurosurgery Service, National Institute of Neurology and Neurosurgery, Mexico City, Mexico; ^4^Faculty of Medicine, National Autonomous University of Mexico, Mexico City, Mexico

**Keywords:** epilepsy, intracranial arteriovenous malformations, neurosurgery, radiosurgery, seizures

## Abstract

Intracranial arteriovenous malformations (AVMs) are vascular anomalies that can present with intracranial hemorrhage, seizures, or neurological deficits. In this case, we present a woman with a giant right frontoparietal AVM (Spetzler–Martin Grade V) initially diagnosed after an intracerebral hemorrhage at Age 6. Surgical, endovascular, and radiosurgical treatments were not viable due to the lesion's size and eloquent location. Over time, the patient developed focal seizures, including catamenial patterns and left-arm spastic monoparesis. Initial antiseizure medications (ASMs) such as carbamazepine and phenytoin failed to provide adequate control at optimal dosage, with phenytoin exacerbating seizure frequency. Partial seizure control was eventually achieved with a combination of levetiracetam and carbamazepine. Neuroimaging showcases a large AVM, while EEG revealed focal epileptiform activity. This case illustrates the complexity of treating epilepsy secondary to giant AVMs, emphasizing the need for individualized ASM strategies and collaborative, multidisciplinary management.

## 1. Introduction

Intracranial arteriovenous malformations (AVMs) are vascular lesions characterized by an arteriovenous shunt formed from a tangle of anomalous vessels without a normal capillary bed [1]. Most lesions reach attention in patients in their 40s, and 75% of the hemorrhagic presentations occur before the age of 50 years, affecting both sexes equally [2]. Cerebral AVMs may present with intracranial hemorrhage, seizures, headaches, and long-term disability [3].

Epileptic seizures are the second most common manifestation of AVMs, which can occur in 20%–45% of patients [4], after intracranial hemorrhage, which is the most frequent and feared component of AVMs. Otherwise, seizures are the most common presentation of unruptured AVMs [5]. Cortical AVMs, particularly those involving the temporal lobe, confer the highest risk for seizures. Other potential risk factors for AVM-associated seizures are larger nidus size, superficial venous drainage, and arterial border zone location [6].

The surrounding brain tissue might be affected by venous congestion owing to high input of blood or reduced outflow. Furthermore, a long pial course of the draining vein might indicate that normal venous drainage is restricted over a large cortical area, which increases the risk of developing seizures [7].

The selection of treatment options for cerebral AVMs is a complex process that must be guided by the specific characteristics of the vascular lesion, including factors such as the size and location of the AVM. Numerous cases of AVMs and their management have been well-documented. However, there is a lack of information regarding AVMs of the exceptional size observed in our case; the objective of this report is to illustrate the extent to which an AVM can reach an extraordinary size, its associated clinical manifestations, and the potential treatment options available for managing such rare cases, as there are not many reported cases of this type.

## 2. Case Presentation

**Figure 1:**
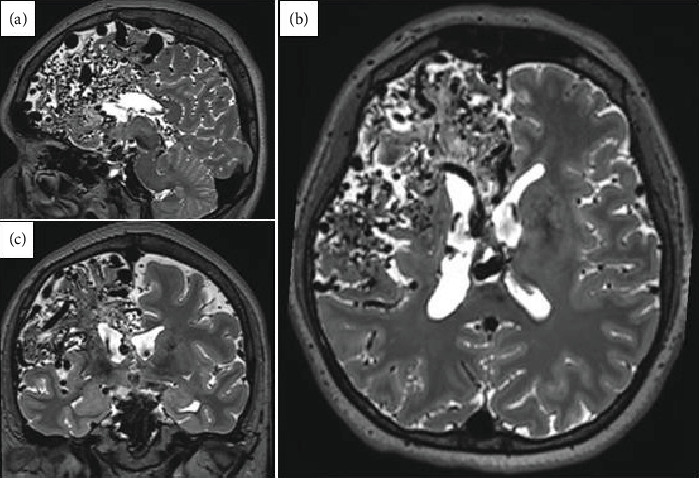
3.0 T brain MRI T2 sequence performed at Age 6 revealing a large 11 cm AVM in the right frontal lobe, extending to the insular and parietal regions. The AVM receives arterial supply from the right middle cerebral artery and the anterior cerebral arteries, compromising the perirolandic region. Additional findings include areas of gliosis and hemosiderin deposition. (a) Sagittal view. (b) Coronal view. (c) Axial view.

A right-handed female in her late 30s with no relevant family and medical history of epilepsy or other neurological disorders. Her neurodevelopmental milestones were achieved without any alterations. At the age of six, the patient presented with an intracerebral hemorrhage, prompting immediate medical evaluation and management of the cerebrovascular event. As part of the diagnostic workup, a brain MRI and CT angiography were performed, revealing a right frontoparietal giant AVM classified as Spetzler–Martin Grade V (Figure 1). Due to the AVM characteristics, the patient was deemed unsuitable for microsurgical resection, embolization, or radiosurgery.

During follow-up, the patient experienced a tonic–clonic seizure and was initially treated with carbamazepine (200 mg/day), achieving partial seizure control. Subsequently, she developed left-arm spastic monoparesis and focal seizures. These seizures began with symptoms of tachycardia and anxiety, followed by paresthesia in the left upper limb lasting approximately 30 s, progressing to facial myoclonus and clonic motor activity involving the left hemibody, accompanied by postictal numbness and fatigue. The seizures exhibited a catamenial pattern, with each episode lasting 1–2 min and occurring without loss of consciousness.

**Figure 2:**
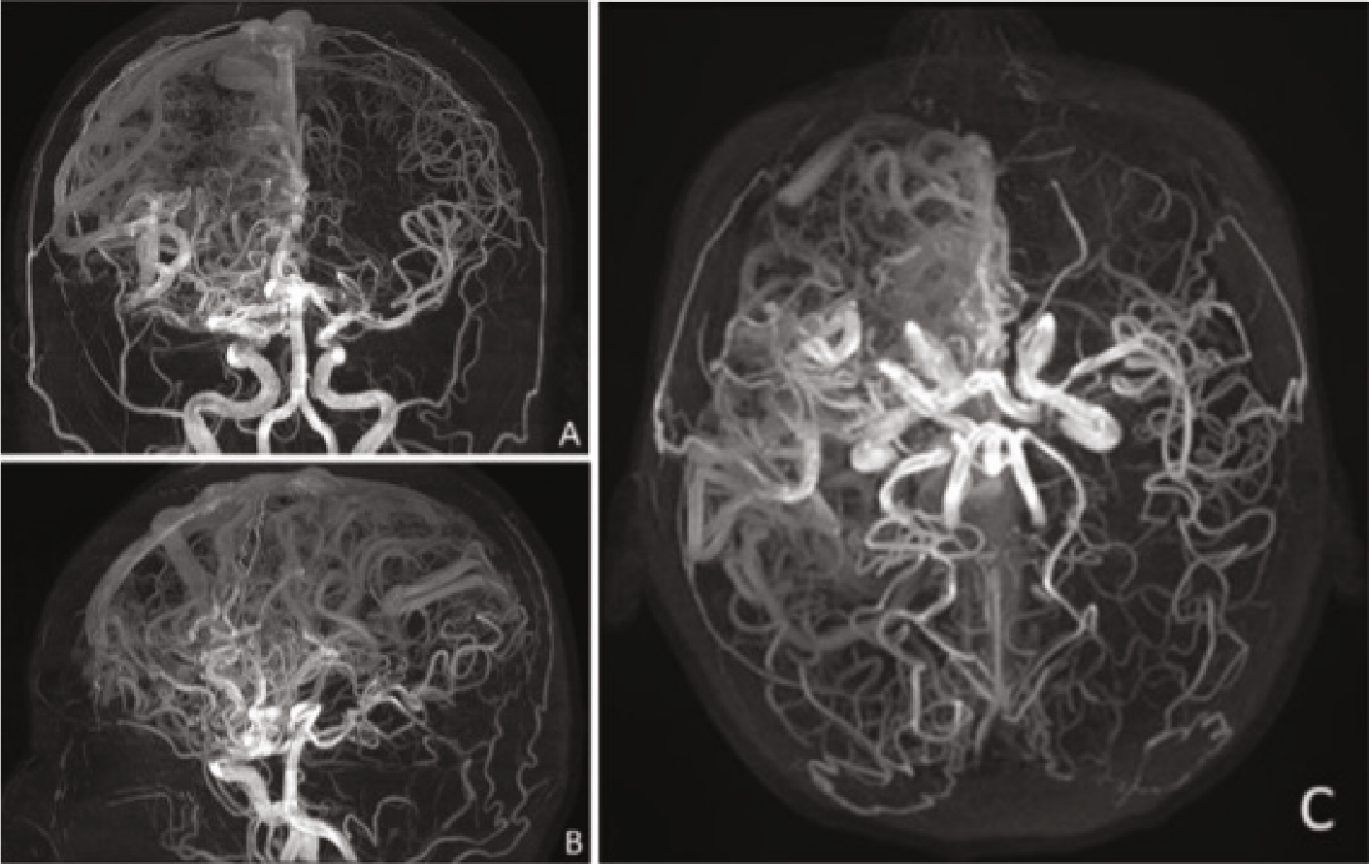
3D angio-MRI illustrating the extensive vascular architecture of the AVM, its lobar distribution, and complex arterial supply. The image highlights the AVM's size and involvement of the right frontal, parietal, and insular lobes. (A) Coronal view. (B) Sagittal view. (C) Axial view.

**Figure 3:**
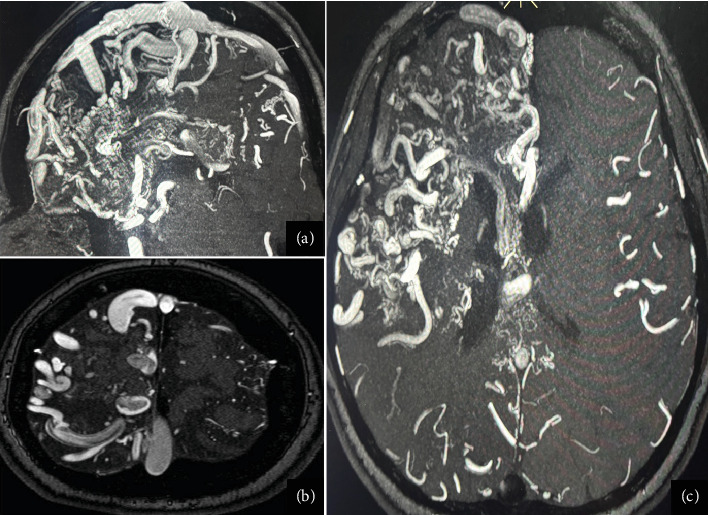
3D time-of-flight MR angiography (3D-TOF MRA) demonstrating the AVM's superficial and deep venous drainage. The image shows convergence of dilated cortical veins and deep venous structures toward the superior sagittal, sphenoparietal, and transverse sinuses, providing imaging evidence consistent with a Spetzler–Martin Grade V classification. (a) Sagittal view. (b) Axial view (superior slice). (c) Axial view (inferior slice).

**Figure 4:**
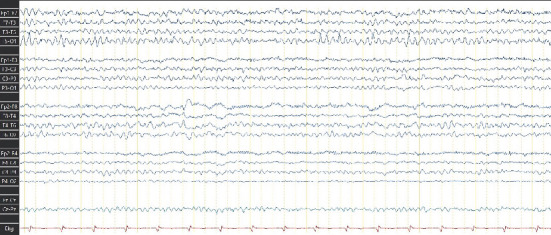
Scalp EEG findings indicating focal slowing and interictal epileptiform activity in the fronto-centrotemporal region and epileptiform activity in the right anterior temporal region.

Given the lack of seizure control, her antiseizure medication (ASM) was switched to phenytoin (300 mg/day), which resulted in no improvement and, in fact, led to an increase in seizure frequency, including seizure clusters on several occasions. Her treatment regimen was subsequently adjusted to include levetiracetam (2 g/day) and carbamazepine (600 mg/day). This combination achieved a reduction in seizure frequency, with the patient currently experiencing approximately one seizure per month. Due to the high risks associated with the Grade V AVMs, the patient is not a candidate for surgical resection, endovascular therapy, or radiosurgery. As part of the most recent follow-up, the patient underwent a comprehensive evaluation. Physical examination still reveals distal spastic paresis in the left arm, and the patient underwent a 3D angio-MRI (Figure 2), which confirmed the persistence of an 11 cm AVM involving the right frontal, parietal, and insular lobes. While this sequence provided a clear depiction of the lesion's size and lobar extension, it did not adequately visualize deep venous drainage. Therefore, a complementary 3D time-of-flight MR angiography (Figure 3) was performed, which successfully demonstrated both superficial and deep venous outflow, including drainage into the superior sagittal, sphenoparietal, and transverse venous sinuses. These findings support the classification of the lesion as a Spetzler–Martin Grade V AVM. Additionally, scalp EEG findings indicated focal slowing and epileptiform activity in the fronto-centrotemporal region (Figure 4). Management is medical only, with partial control of her epileptic seizures.

## 3. Discussion

We present the case of a young adult patient diagnosed with a giant AVM following a cerebrovascular event. The patient subsequently developed epilepsy, most likely triggered by the initial hemorrhage, as AVM rupture is a well-established risk factor for seizures and epilepsy. However, the pathophysiology of seizures in the context of AVMs is multifactorial [6]. In this case, the seizures may have resulted directly from the hemorrhage or from structural brain changes associated with vascular steal phenomena, or even due to the size and location of the AVM nidus. Notably, AVM location has been identified as a significant predictor of seizure onset. Zhang et al. [8] reported that seizure occurrence and the development of epilepsy are more closely associated with damage to specific brain regions, particularly the right precentral gyrus and the right superior longitudinal fasciculus. These findings are consistent with the imaging and clinical features observed in our patient.

According to available evidence on the use of ASMs for structural epilepsy secondary to AVMs, there is no specific ASM recommended for seizure control. Patients with primary generalized seizures require different antiepileptic drugs (AEDs) compared to those with focal seizures. As treatment recommendations evolve over time, the involvement of an epileptologist is advisable for optimal management [9].

In cases of frequent seizures or drug-resistant epilepsy (DRE), consideration of epilepsy surgery by vascular surgeons becomes essential. Spetzler and Martin introduced a grading system for AVMs in 1986, which evaluates size, location, and venous drainage patterns and remains a cornerstone in clinical decision-making [10].

Grade V AVMs in the Spetzler–Martin classification are characterized by a size exceeding 6 cm, deep venous drainage, and localization within eloquent brain regions. These lesions are generally not suitable for microsurgical resection and are often managed conservatively when asymptomatic. However, progressive neurological deficits, recurrent hemorrhages, or persistent seizures may necessitate intervention, regardless of the AVMs' size and location [9, 11].

Although the pathogenesis of AVMs and their role in associated epilepsy remain unclear, clinical studies have demonstrated that their obliteration following microsurgical treatment and stereotactic radiosurgery (SRS) is currently the most successful approach in terms of seizure reduction rates [12, 13]. It is important to note that a considerable percentage of seizure reduction following SRS is not directly proportional to the extent of residual arteriovenous interruption. Regis et al. have proposed the neuromodulatory effect of ionizing radiation and its functional role in the reorganization of networks within the microarchitecture adjacent to the AVM [14].

Regarding seizure management in patients with AVM-related epilepsy, the approach remains controversial. Large trials and prospective studies have shown a lack of significant changes in seizure reduction or an increase in seizure frequency when comparing interventional management with expectant medical management [15–18].

A review by Alkhalili et al. included patients with large AVMs (Spetzler–Martin Grades IV and V), where seizures were the most common presenting symptom. These patients underwent staged volume radiosurgery (SVR), which has shown promise in treating AVMs, achieving satisfactory obliteration rates while minimizing damage to surrounding brain tissue. Despite these advantages, SVR outcomes are influenced by several challenges, and the role of adjunctive embolization remains controversial in managing large AVMs [19].

Ironside et al. published a meta-analysis that reported seizure outcomes in a total of 4826 patients across 27 studies who underwent SRS for AVM-associated seizures. Seizure control (seizure freedom or seizure improvement) was achieved in 910 of 1312 patients (random effects model, 73.1% [95% CI 66.9%–78.9%]), with complete seizure freedom being achieved in 597 of 1245 patients (random effects model, 55.7% [95% CI 44.5%–66.6%]). Seizure status remained unchanged in 219 of 914 patients (random effects model, 18.7% [95% CI 12.4%–26.0%]) and worsened in 39 of 675 patients (random effects model, 6.1% [95% CI 3.3%–9.7%] [20].

As we have reviewed, the management of giant AVMs with epilepsy remains complex and multifaceted. While microsurgical resection and SRS have shown promising outcomes in seizure reduction, we opted to continue medical treatment for our patient, resulting in moderate improvement in both her seizure frequency and overall well-being at present.

## 4. Conclusions

Although it has not been determined whether medical treatment alone is sufficient to control epilepsy associated with AVMs, the safety and efficacy of interventional therapies are well-documented. However, due to the unique clinical presentation, the choice of intervention should be individualized based on the characteristics of the AVMs and the patient's specific clinical situation.

## Data Availability

The data that support the findings of this study are available on request from the corresponding author. The data are not publicly available due to privacy or ethical restrictions.
